# Familial chylomicronemia syndrome due to a heterozygous deletion of the chromosome 8 treated with the apoCIII inhibitor volanesorsen: A case report

**DOI:** 10.1097/MD.0000000000027573

**Published:** 2021-10-22

**Authors:** Adrienn Tünnemann-Tarr, Hubert Scharnagl, Julius L. Katzmann, Paulina Stürzebecher, Ulrich Laufs

**Affiliations:** a Klinik und Poliklinik für Kardiologie, Universitätsklinikum Leipzig, Leipzig, Germany; b Klinisches Institut für Medizinische und Chemische Labordiagnostik, Medizinische Universität Graz, Graz, Austria.

**Keywords:** apolipoprotein CIII inhibitor, familial chylomicronemia syndrome, hypertriglyceridemia, pancreatitis, volanesorsen

## Abstract

**Rationale:**

Familial chylomicronemia syndrome is a congenital, severe form of hypertriglyceridemia associated with increased risk of acute pancreatitis. Treatment options are limited.

**Patient concerns:**

A 52-year-old woman was referred with recurrent pancreatitis and severe hypertriglyceridemia to our lipid clinic.

**Diagnosis:**

Laboratory examination showed elevated serum triglyceride concentrations of 8090 mg/dL (90 mmol/L). Lipid electrophoresis showed a type V phenotype with positive chylomicrons. Genetic investigation revealed a novel heterozygous large deletion of the lipoprotein lipase gene on chromosome 8. A familial chylomicronemia syndrome was diagnosed. Other causes of hypertriglyceridemia were excluded.

**Interventions:**

Fibrates and diet did not lower triglyceride levels. Therefore, treatment with the apolipoprotein CIII (apoCIII) inhibitor volanesorsen was initiated.

**Outcomes:**

After 3 months of treatment, a 90% reduction of triglycerides was observed. ApoCIII concentrations were reduced by 90% in the total and by 61% in the chylomicron-free serum. Treatment was well tolerated with only minor local reaction after the first application. The platelet count was monitored weekly and did not decrease <150 cells/μL.

**Lessons:**

This case report shows that inhibition of apoCIII potently reduces serum triglycerides in patients with heterozygous monogenetic deletion of the lipoprotein lipase gene. Follow-up will show the effect on recurrent episodes of pancreatitis.

## 1 Introduction

Familial chylomicronemia syndrome (FCS) is a congenital, severe form of hypertriglyceridemia, caused by mutations of the lipoprotein lipase (*LPL*) gene.^[1]^ LPL catalyzes lipolysis in triglyceride-rich lipoproteins such as chylomicrons. LPL deficiency leads to highly elevated triglyceride and chylomicron serum concentrations. FCS is associated with an increased risk of acute pancreatitis, a potentially life-threatening disease. The diagnosis of FCS is often delayed until adulthood.^[2]^ The estimated prevalence of the FCS is 1 to 10 per million.^[3]^ We report a case of FCS that is of interest because of a novel mutation, a heterozygote large deletion within the chromosome 8, and the effects of treatment with the apolipoprotein CIII (apoCIII) inhibitor volanesorsen on serum lipoproteins.

## 2 Case report

In 2019, a 52-year-old woman presented to our lipid clinic. During the previous year, she was hospitalized twice with severe acute pancreatitis. The patients’ medical history included a history of breast cancer on the left side, treated with ablation and radio- and chemotherapy. The patient had a well-controlled diabetes mellitus type 2 with an HbA1c of 6.3%. The son of the patient has elevated triglyceride levels.

**Figure 1:**
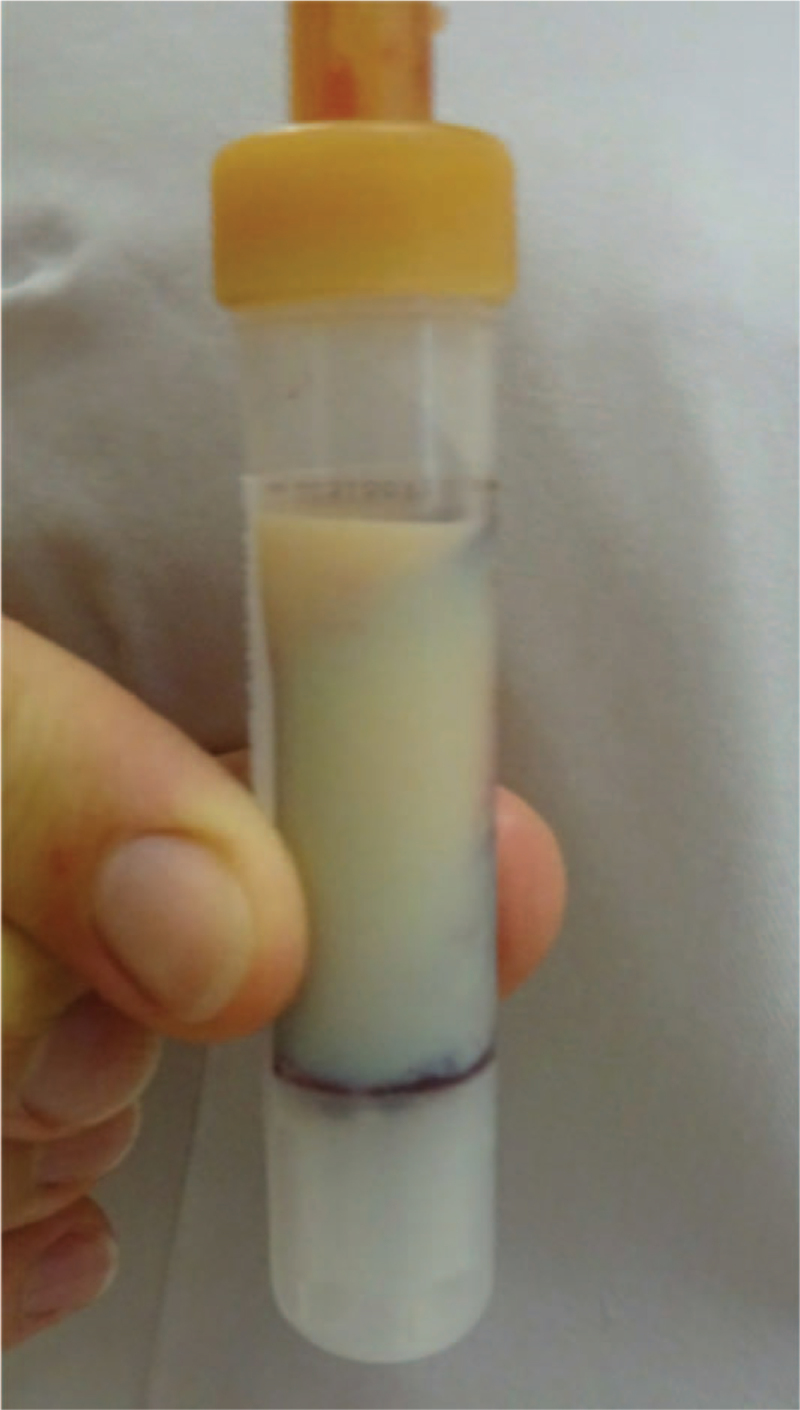
Positive refrigerator test. Shown is a serum specimen stored 24 hours in a refrigerator. In the presence of chylomicrons, these build a creamy layer at the top of the specimen, while the serum becomes clear; in contrast, very-low density lipoprotein remains dissolved in the serum.

Laboratory examination showed a markedly elevated triglyceride level of 8090 mg/dL (90 mmol/L). The refrigerator test was positive (Fig. 1). The lipoprotein electrophoresis showed a type V hyperlipoproteinemia phenotype with positive chylomicrons. Other secondary factors contributing to hypertriglyceridemia (such as alcohol consumption, insufficient diet, renal insufficiency, metabolic syndrome, medical induced hypertriglyceridemia, thyroid disease) were excluded.

The patient had no signs of atherosclerotic disease, the intima-media thickness of the common carotid artery was normal with 0.6 mm on both sides.

**Figure 2:**
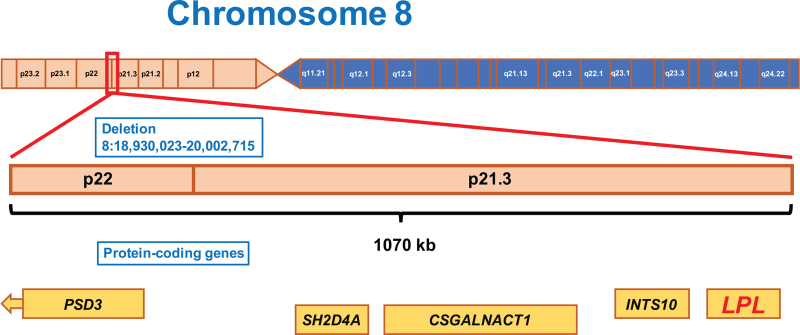
The deletion within chromosome 8. Shown is the location of the novel, large mutation encompassing 1070 kb.

The FCS score was 10 (positive).^[4]^ Genetic investigation revealed a previously not described heterozygous deletion of the chromosome 8 (Chr,8:18,930,023–20,002,715) (Fig. 2). This large deletion is 1070 kb long and involves 5 protein-coding genes including the whole LPL protein-coding gene explaining the remarkable chylomicronemia.

The patient followed a low fat and low carbohydrate diet. In spite of the usage of bezafibrate for several years, the triglyceride levels did not show any changes and the second episode of severe pancreatitis happened during the fibrate treatment.

To prevent recurrent pancreatitis, treatment with volanesorsen, an antisense oligonucleotide inhibitor of apoCIII, was initiated with a weekly dose of 285 mg s.c. for the first 3 months.^[5]^

**Figure 3:**
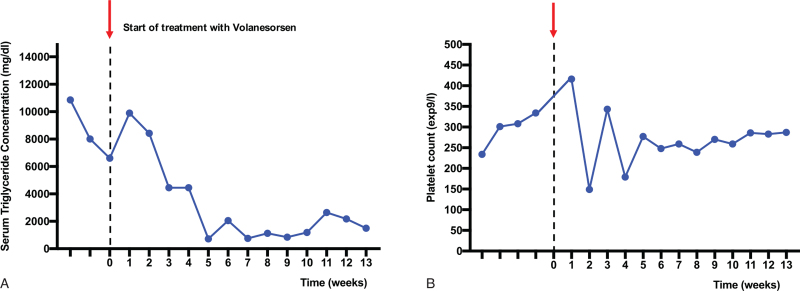
A: Serum triglycerides during the treatment with volanesorsen. Shown are the triglyceride serum concentrations over time, with markedly reductions after initiation of treatment with volanesorsen. B: Platelet count during the treatment with volanesorsen. Shown is the platelet count over time, with no occurrence of relevant thrombozytopenia.

Lipoprotein separation and analysis was performed from frozen serum samples (stored at −80°C). Lipids and apolipoproteins were measured in fasting total serum and after ultracentrifugation in chylomicron-free serum. Lipids and apolipoproteins in chylomicrons were calculated as difference between serum and chylomicron-free serum. ApoCIII levels showed a 90% decrease in the total serum (120 mg/dL vs 12 mg/dL) and 61% in chylomicron free serum (44 mg/dL vs 17 mg/dL). In the chylomicron fraction, a 96% reduction of the triglyceride level was observed and apoCIII was not measurable (Table 1). Within 3 months, a 90% reduction of triglyceride concentration was observed, with a lowest value of 721 mg/dL (Fig. 3A). In parallel to the decrease of chylomicrons, the concentrations of cholesterol and apolipoprotein B in very low-density lipoproteins (VLDL) and low-density lipoprotein (LDL) increased (Table 1), indicating that lipolysis and the production of cholesterol-rich particles were partially recovered. The treatment was well tolerated. Local reaction occurred only after the first application of the subcutaneous injection. Weekly monitoring showed a mild decrease of the platelet count that was transient (Fig. 3B).^[6]^

**Table 1:** Lipids and apolipoproteins in fasting serum and after ultracentrifugation (chylomicron-free serum and in the chylomicron fraction) during the treatment with volanesorsen.

	Start of the treatment with volanesorsen	Week 1	Week 2	Week 6	Week 9
Serum with chylomicrons					
Cholesterol	1292	1285	1315	539	293
Triglycerides	6603	9899	8421	2052	841
ApoCIII	120	133	106	25	12
Serum after separation of chylomicrons					
Cholesterol	65	88	125	86	195
Triglycerides	276	482	640	197	448
ApoCIII	44	32	40	26	17
Chylomicrons					
Cholesterol	1227	1197	1190	453	98
Triglycerides	6327	9417	7781	1855	393
ApoCIII	76	101	66	ND	ND
VLDL					
VLDL-cholesterol	30	58	95	23	100
VLDL-apoB	7	17	21	9	37
LDL					
LDL-cholesterol	25	18	18	48	71
LDL-apoB	36	44	45	62	73

## 3 Discussion

FCS is characterized by fasting blood triglyceride concentrations >885 mg/dL (>10 mmol/L) due to chylomicronemia.^[4]^ Chylomicrons are large lipoproteins formed in the intestine during the absorption of dietary fats. In conditions of reduced activity of their primary degrading enzyme, the LPL, chylomicron degradation is impaired causing elevated and persisting serum triglyceride levels.

Symptoms of FCS include severe abdominal pain with or without pancreatitis, leading to metabolic complications and frequent hospitalizations. The typical and most serious complication of chylomicronemia is acute pancreatitis. In the majority of FCS patients, the episodes occur recurrently. Some of the patients develop chronic pancreatitis that can lead to pancreoprive diabetes mellitus (type 3).^[6]^ One episode of acute pancreatitis is able to cause permanent damage to the pancreas which is associated with a mortality rate of 6%.^[7]^

Two mechanisms contribute to the development of pancreatitis. The accumulation of chylomicrons reduces the blood flow to the pancreas, which leads to ischemia.^[8]^ In addition, the triglycerides are degraded to free fatty acids by the pancreatic lipase. The clogged chylomicrons cause acinar damage and exposure of triglycerides to the pancreatic lipase. Due to the release of free fatty acids, acinar damage occurs. The conversion of trypsinogen and trypsin releases inflammatory cytokines. Cytotoxic injury occurs, resulting in an increase in inflammatory mediators and free radicals.^[8]^ The risk of pancreatitis increases with the higher serum triglycerides. Patients with FCS have a 360-fold increased risk of acute pancreatitis.^[9]^

Additional symptoms of patients with FCS include eruptive xanthomas, arthralgias, lipemia retinalis or hepatosplenomegaly.^[9]^ In addition, there are neurological symptoms such as mild dementia, confusion, memory loss and fatigue. FCS is associated with emotional burden on the patients and their relatives, which can lead to anxiety, feelings of guilt, social isolation, and depression.^[10]^ The social isolation is amplified by the special dietary requirements.

Affected patients usually do not respond to conventional triglyceride-lowering therapies. In the past, treatment of FCS patients was limited to very strict dietary fat restriction and avoidance of alcohol and certain medications.^[3,11]^ Plasmapheresis has been tried in some FCS cases.^[12]^ In the presence of diabetes, intravenous administration of insulin is required. The extremely restrictive diet and the risk of recurrent pancreatitis limits the quality of life. The compliance with the diet regimen over long periods is challenging.^[13]^

FCS is caused by a rare gene defect that is currently known to affect *LPL*, *APOC2*, *GPIHBP1*, *APOA5*, and *LMF1*. Mutations in the *LPL* gene are responsible for the development of 80% of cases. LPL is a key enzyme in the catalysis of triglycerides. Reduced LPL function causes chylomicronemia. Until now, 5 deletions of the *LPL* gene have been described in the literature.^[14]^ The new deletion reported in this patient for the first time is hundred times larger than the previously described alterations which likely explains the severe clinical manifestation in our patient.^[14,15]^

One of the proteins affecting LPL activity is apoCIII.^[16]^ ApoCIII inhibits LPL activity, reduces the hepatic uptake of triglyceride-rich lipoproteins and increases hepatic secretion of triglycerides.^[17]^ Loss-of-function (LOF) mutations of the gene coding for apoCIII and pharmacologic inhibition of apoCIII lead to reduced plasma triglyceride levels.^[18]^ The novel drug volanesorsen selectively binds within the 3′ untranslated region of apoCIII messenger ribonucleic acid (mRNA). This binding allows mRNA degradation prevents the translation of the apoCIII mRNA and lowers triglycerides.^[19]^

In our patient, volanesorsen rapidly reduced apoCIII serum concentrations and serum triglyceride concentration by 90%. In parallel to the observed reduction of chylomicrons, the concentrations of cholesterol and apolipoprotein B in VLDL and LDL increased showing partial restoration of lipolysis and of cholesterol-rich particles. The treatment was very well tolerated. The only adverse effect was a mild and transient decrease of the platelet count.

In summary, patients with FCS and recurrent pancreatitis have a very high morbidity with limited treatment options.^[20]^ This case identifies a novel mutation causing severe FCS and is an example of a novel treatment modality using an RNA-inhibiting drug targeting apoCIII . Prospective studies with clinical endpoints are wanted.

## Author contributions

AT and UL wrote the article, AT, JLK, PS, and UL diagnosed and treated the patient, HS performed the laboratory analyses. All authors approved the final article.

**Conceptualization:** Adrienn Tünnemann-Tarr, Julius L. Katzmann, Ulrich Laufs.

**Data curation:** Adrienn Tünnemann-Tarr, Julius L. Katzmann.

**Investigation:** Adrienn Tünnemann-Tarr, Hubert Scharnagl, Julius L. Katzmann, Paulina Stürzebecher, Ulrich Laufs.

**Methodology:** Adrienn Tünnemann-Tarr, Hubert Scharnagl, Ulrich Laufs.

**Project administration:** Ulrich Laufs.

**Supervision:** Ulrich Laufs.

**Validation:** Ulrich Laufs.

**Visualization:** Adrienn Tünnemann-Tarr, Julius L. Katzmann.

**Writing – original draft:** Adrienn Tünnemann-Tarr, Ulrich Laufs.

**Writing – review & editing:** Adrienn Tünnemann-Tarr, Hubert Scharnagl, Julius L. Katzmann, Paulina Stürzebecher, Ulrich Laufs.

## References

[1] Williams L Rhodes KS Karmally W Welstead LA Alexander L Sutton L. Familial chylomicronemia syndrome: bringing to life dietary recommendations throughout the life span. J Clin Lipidol 2018;12:908–19. 29804909 10.1016/j.jacl.2018.04.010

[2] Baass A Paquette M Bernard S Hegele RA. Familial chylomicronemia syndrome: an under-recognized cause of severe hypertriglyceridaemia. J Intern Med 2020;287:340–8. 31840878 10.1111/joim.13016

[3] Laufs U Parhofer KG Ginsberg HN Hegele RA. Clinical review on triglycerides. Eur Heart J 2020;41:99–109c. 31764986 10.1093/eurheartj/ehz785 PMC6938588

[4] Moulin P Dufour R Averna M. Identification and diagnosis of patients with familial chylomicronaemia syndrome (FCS): Expert panel recommendations and proposal of an “FCS score”. Atherosclerosis 2018;275:265–72. 29980054 10.1016/j.atherosclerosis.2018.06.814

[5] Feingold KR. Endotext: Triglyceride Lowering Drugs. South Dartmouth (MA); 2000.

[6] Gaudet D Brisson D Tremblay K. Targeting APOC3 in the familial chylomicronemia syndrome. N Engl J Med 2014;371:2200–6. 25470695 10.1056/NEJMoa1400284

[7] Zhang R Deng L Jin T. Hypertriglyceridaemia-associated acute pancreatitis: diagnosis and impact on severity. HPB (Oxford) 2019;21:1240–9. 30885545 10.1016/j.hpb.2019.01.015

[8] Guo YY Li HX Zhang Y He WH. Hypertriglyceridemia-induced acute pancreatitis: progress on disease mechanisms and treatment modalities. Discov Med 2019;27:101–9. 30939294

[9] Brunzell JDBE Bierman EL. Chylomicronemia syndrome. Interaction of genetic and acquired hypertriglyceridaemia. Med Clin North Am 1982;66:455–68. 7040847 10.1016/s0025-7125(16)31430-4

[10] Davidson M Stevenson M Hsieh A. The burden of familial chylomicronemia syndrome: Results from the global IN-FOCUS study. J Clin Lipidol 2018;12:898e.2–907.e2. 29784572 10.1016/j.jacl.2018.04.009

[11] Zheng Z Ding Y-X Qu Y-X Cao F Li F. A narrative review of acute pancreatitis and its diagnosis, pathogenetic mechanism, and management. Ann Transl Med 2021;9:69. 33553362 10.21037/atm-20-4802 PMC7859757

[12] Chang C-T Tsai T-Y Liao H-Y. Double filtration plasma apheresis shortens hospital admission duration of patients with severe hypertriglyceridemia-associated acute pancreatitis. Pancreas 2016;45:606–12. 26491906 10.1097/MPA.0000000000000507

[13] Hegele RA Borén J Ginsberg HN. Rare dyslipidaemias, from phenotype to genotype to management: a European Atherosclerosis Society task force consensus statement. Lancet Diabetes Endocrinol 2020;8:50–67. 31582260 10.1016/S2213-8587(19)30264-5

[14] Dron JS Wang J McIntyre AD. Partial LPL deletions: rare copy-number variants contributing towards severe hypertriglyceridemia. J Lipid Res 2019;60:1953–8. 31519763 10.1194/jlr.P119000335 PMC6824486

[15] Iacocca MA Dron JS Hegele RA. Progress in finding pathogenic DNA copy number variations in dyslipidemia. Curr Opin Lipidol 2019;30:63–70. 30664016 10.1097/MOL.0000000000000581

[16] West G Rodia C Li D Johnson Z Dong H Kohan AB. Key differences between apoC-III regulation and expression in intestine and liver. Biochem Biophys Res Commun 2017;491:747–53. 28739253 10.1016/j.bbrc.2017.07.116 PMC6069593

[17] Borén J Packard CJ Taskinen M-R. The roles of ApoC-III on the metabolism of triglyceride-rich lipoproteins in humans. Front Endocrinol (Lausanne) 2020;11:474. 32849270 10.3389/fendo.2020.00474 PMC7399058

[18] Taskinen M-R Packard CJ Borén J. Emerging evidence that ApoC-III inhibitors provide novel options to reduce the residual CVD. Curr Atheroscler Rep 2019;21:27. 31111320 10.1007/s11883-019-0791-9 PMC6527792

[19] Witztum JL Gaudet D Freedman SD. Volanesorsen and triglyceride levels in familial chylomicronemia syndrome. N Engl J Med 2019;381:531–42. 31390500 10.1056/NEJMoa1715944

[20] Garg R Rustagi T. Management of hypertriglyceridemia induced acute pancreatitis. Biomed Res Int 2018;2018:4721357. 30148167 10.1155/2018/4721357 PMC6083537

